# Hypervirulent clade 2, ribotype 019/sequence type 67 *Clostridioides difficile* strain from Japan

**DOI:** 10.1186/s13099-019-0336-3

**Published:** 2019-11-04

**Authors:** Ryoichi Saito, Yukino Usui, Alafate Ayibieke, Jun Nakajima, Isaac Prah, Kazunari Sonobe, Yoshibumi Aiso, Shiori Ito, Yasuhiro Itsui, Yoshiro Hadano, Yoko Nukui, Ryuji Koike, Shuji Tohda

**Affiliations:** 1 Department of Molecular Microbiology, Graduate School of Medical and Dental Sciences, Tokyo Medical and Dental University, Tokyo, Japan; 2 Department of Infection Control and Prevention, Tokyo Medical and Dental University Medical Hospital, Tokyo, Japan; 3 Department of Clinical Laboratory, Tokyo Medical and Dental University Medical Hospital, Tokyo, Japan; 4 Department of Gastroenterology and Hepatology, Tokyo Medical and Dental University Medical Hospital, Tokyo, Japan

**Keywords:** *Clostridioides difficile*, Hypervirulent clade 2, Ribotype 019, Sequence type 67, Toxin A, Toxin B, Binary toxin, Pathogenicity locus, Cdt locus

## Abstract

**Background:**

*Clostridioides difficile* ribotype (RT) 019/sequence type (ST) 67 strains belong to a hypervirulent lineage closely related to RT027/ST1; however, limited data are available for hypervirulent clade 2 lineages in Japan. Herein, we report the draft genome of a *C. difficile* strain B18-123 belonging to clade 2, RT019/ST67 for the first time in Japan.

**Results:**

The pathogenicity locus carried by B18-123 (19.6 kb) showed higher homology (97.29% nucleotide identity) with strain R20291 (RT027/ST1) than the reference strain 630 (RT012/ST54), and B18-123 harbored 8-nucleotide substitutions in *tcdC*. However, it did not contain an 18-base pair (bp) deletion or a single-bp deletion at position 117 in *tcdC*, which was identified in the previous strain R20291. A cytotoxicity assay revealed similar cytotoxicity levels between strains B18-123 and ATCC BAA-1870 (RT027/ST1). The B18-123 strain was found to be susceptible to metronidazole and vancomycin.

**Conclusion:**

Our findings contribute to the further understanding of the characteristics of hypervirulent clade 2 including RT019/ST67 lineages.

## Introduction

*Clostridioides difficile* infection (CDI) is a well-known cause of healthcare-associated infections, ranging from mild diarrhea to severe pseudomembranous colitis and even death. It may be related to disruption of the indigenous intestinal microbiota by prolonged treatment with drugs such as antibiotics [1]. The incidence of CDI has increased over the past decade and clinical manifestations are typically triggered by toxin A (TcdA) and toxin B (TcdB) [2].

Epidemic hypervirulent strains with binary toxin (Cdt), North American pulsed-field type one (NAP1)/ribotype (RT) 027/sequence type (ST) 1 belonging to clade 2 and RT078/ST11 belonging to clade 5 have been identified as major lineages responsible for outbreaks in North America and Europe because of their high sporulation capacity and toxin production and resistance to fluoroquinolones [3–6]. However, these strains are rarely reported in Asia including in Japan, where other lineages such as RT014, RT017, and RT018 have been known to be predominant [7, 8]. RT019/ST67, which is closely related to NAP1/RT027/ST1 among clade 2 [9], produces high levels of toxins upon *tcdC* repression caused by an 18-base pair (bp) deletion and a single-bp deletion at position 117 in *tcdC* [10]. Although the previously reported RT019/ST67 strains [10–12] have not shown any resistance to vancomycin and metronidazole, additional data are needed to understand the characteristics of the RT019/ST67 lineage to implement more effective treatments and methods of infection control.

In Japan, NAP1/RT027/ST1 strains are occasionally isolated from patients with fulminant colitis [13, 14]. However, limited data, including genome sequences, are available for hypervirulent clade 2 because of its extremely low prevalence among CDI incidences in Japan [15]. We reported the detection of a *C. difficile* strain B18-123 belonging to hypervirulent clade 2, RT019/ST67, in Japan isolated from a patient with multiple recurrent episodes of severe diarrhea.

## Methods

### Bacterial isolates and growth conditions

*Clostridioides difficile* strain B18-123 was obtained from a stool sample of a 81-year-old elderly male patient who underwent chemotherapy for hepatocellular carcinoma at Medical Hospital of Tokyo Medical and Dental University in 2018. Bacterial identification was performed using the C. DIFF QUIK CHEK COMPLETE test (Abbott Diagnostics Medical, Tokyo, Japan) and confirmed by 16S rRNA gene sequencing [16] for quality assurance of the following data. *C. difficile* strains American Type Culture Collection (ATCC) 700057 (RT038/ST48; TcdA^−^, TcdB^−^, Cdt^−^) and ATCC BAA-1870 (NAP1/RT027/ST1; TcdA^+^, TcdB^+^, Cdt^+^) were also used in the study. The *C. difficile* strains were grown at 37 °C under anaerobic conditions using brain heart infusion (BHI; Oxoid, Basingstoke, UK) broth or agar plate supplemented with 5 g/L yeast extract and 0.1% (w/v) l-cysteine (BHIS).

### Multilocus sequence typing, ribotyping, and toxin profiles

Multilocus sequence typing (MLST) and ribotyping were performed as previously described [16, 17]. *tcdA*, *tcdB*, *cdtA*, and *cdtB* were detected by multiplex PCR [18], followed by analysis of the length of *tcdA* as described [19]. A cytotoxicity assay was performed for B18-123 together with the ATCC BAA-1870 and ATCC 700057 strains as described previously [16].

### Genome sequencing, de novo assembly, and annotation

Genomic DNA was extracted with a NucleoSpin Tissue kit (Takara Bio, Shiga, Japan) and subjected to quality control by agarose gel electrophoresis. The DNA obtained was quantified with a Synergy H1 (BioTek, Winooski, VT, USA) and QuantiFluor dsDNA System (Promega, Madison, WI, USA), and sequenced using a NextSeq platform (Illumina, Inc., San Diego, CA, USA) using a 2 × 151-bp paired-end approach. After quality control on raw reads was performed using Sickle (https://github.com/najoshi/sickle), the reads were assembled de novo using SPAdes version 3.10.1. Gene annotation was determined with Prokka version 1.11. The draft genome was subjected to in silico analyses, such as MLST using MLST version 2.0 [20] and detection of antibiotic resistance mechanisms using ResFinder version 3.1 [21], ARG-ANNOT [22], and CARD [23].

### Comparative genomics

Phylogenetic analysis was performed with a pathogenicity locus (PaLoc) and *tcdC* sequences of 12 complete and draft genomes, including nine ST67 strains deposited in the GenBank, by the maximum likelihood method based on the Tamura-Nei model [24] using MEGA X software [25]. Sequence comparisons of PaLoc and Cdt locus (CdtLoc) were conducted using ClustalW (https://clustalw.ddbj.nig.ac.jp/) and Easyfig version 2.2.2 [26]. The presence of insertion sequences and tRNAs were identified on the PaLoc and its flanking region of B18-123 using ISfinder [27] and tRNAscan-SE [28], respectively. The presence of phage-related sequences was investigated in the whole genome of B18-123 using PHASTER [29].

### Antimicrobial susceptibility testing

The minimum inhibitory concentrations (MICs) of six antibiotics (metronidazole, vancomycin, rifampin, erythromycin, clindamycin, and moxifloxacin) were determined using an Etest (bioMérieux, Marcy-l’Étoile, France) and the breakpoints were based on EUCAST epidemiological cut-off values (http://www.eucast.org/clinical_breakpoints/).

### Statistical analysis

One-way analysis of variance was performed using GraphPad Prism version 7 software to determine the statistical significance of the differences for comparison. p < 0.05 was considered to indicate significance.

### Results and discussion

Following draft genome sequencing of B18-123, a total of 4,284,537 reads were assembled into 98 contigs larger than 200 bp (largest scaffold = 526,102 bp; *N*50 = 277, 155 bp; *L*50 = 5; average GC = 28.39%) with an estimated genome size of 4,221,572 bp and average coverage of 50 ×. MLST analysis in silico along with conventional PCR assigned *C. difficile* strain B18-123 to ST67, which belongs to hypervirulent clade 2. B18-123 strain was classified into RT019 by ribotyping. Although in Japan, only two ST67 strains without ribotype data have been deposited in the GenBank in 2019 (last accessed on September 22, 2019), no report of CDI incidences with RT019/ST67 in Japan has been published so far in English literature. Therefore, our results indicate that a hypervirulent strain with RT019/ST67 responsible for severe diarrhea has been reported for the first time in Japan.

**Fig. 1:**
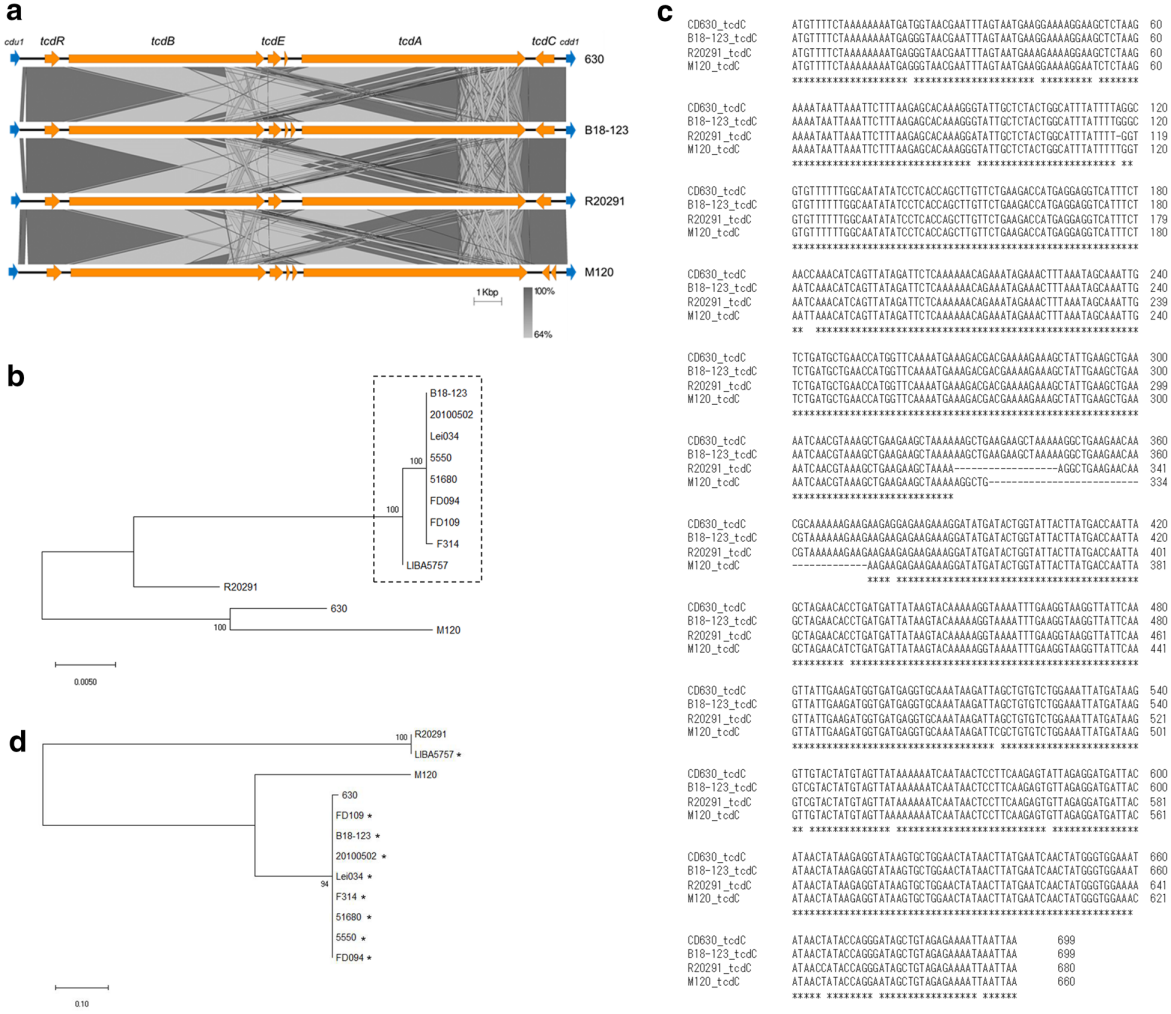
Genetic characteristics of PaLoc in *Clostridioides difficile* strain B18-123. (**a**) Comparative genomic structure analysis of PaLoc among *C. difficile* strains used in this study. (**b**) Phylogenetic tree of PaLoc among 12 strains deposited in the GenBank by maximum likelihood method based on Tamura-Nei model [24] using MEGA X software [25]. Strains surrounded by dotted line belong to ST67. (**c**) *tcdC* sequence alignment for *C. difficile* strains used in this study. Identical nucleotides among their strains are indicated with an asterisk. (**d**) Phylogenetic tree of *tcdC* sequences among 12 strains deposited in the GenBank by maximum likelihood method based on Tamura-Nei model [24] using MEGA X software [25]. Strains indicated with an asterisk belong to ST67

Strain B18-123 possesses *tcdA* and *tcdB*, and their presence was supported by in silico analysis. PaLoc carried by B18-123 was located between two conserved genes, *cdu1* and *cdd1* (Fig. 1a). BLAST results indicated that the PaLoc in B18-123 (19.6 kb) was homologous with the reference genome 630 (accession number AM180355; RT012/ST54; 99% query cover and 94.88% identity), R20291 (accession number FN545816; NAP1/RT027/ST1; 99% query cover and 97.29% identity), and M120 (accession number NC_017174; classified as clade 5, RT078/ST11; 99% query cover and 94.02% identity) strains. This indicates that the PaLoc carried by B18-123 is genetically closely related to that of R20291. These findings were supported by phylogenetic tree analysis of PaLoc carried by 630, R20291, M120, B18-123, and ST67 strains with complete PaLoc deposited in the GenBank (last accessed on September 22, 2019) (Fig. 1b).

In this study, no intact insertion sequences were found 10 kb upstream and downstream from PaLoc; however, truncated sequences (≤ 20 bp) of many insertion sequence families matched with our sequence. These results agree with a previous finding that *C. difficile* PaLoc does not fit the genetically accepted definition of pathogenicity islands containing direct repeats and insertion sequences [30]. However, the transfer of a chromosomal fragment containing the PaLoc proved to be caused by a chromosomally integrated mobile genetic element such as conjugative transposon [31, 32]. The detailed mechanism of this transfer has not yet been clarified.

Although no tRNA was identified on the scaffold containing PaLoc (location on scaffold of NODE013; 119,372 bp), 76 tRNAs representing all 20 amino acids were identified in the whole genome of B18-123. As the reference strain 630 and strain R20291 possess 87 tRNAs and 65 tRNAs, respectively, further investigations considering the entire cellular population of tRNAs may reveal the functional role of tRNAs against cellular physiological process, such as modulation of toxin production.

Additionally, although strain B18-123 harbored an 8-nucleotide substitution in *tcdC*, a negative regulator of *tcdA* and *tcdB* [33] compared to the reference genome 630 (Fig. 1c), only a single amino acid substitution of Asp7Glu (caused by a nucleotide T21G substitution), which was located outside of the three predicted domains [34], was found in 232 amino acids among both strains. Moreover, we did not detect an 18-bp deletion or a single-bp deletion at position 117, or a 39-bp deletion in *tcdC* as previously reported [3, 10, 11, 33, 35, 36]. On the other hand, we found that the *tcdC* sequences of ST67 strains (20100502, Lei034, F314, 5550, 51680, FD094, and FD109), except for strain LIBA5757 [10], were 100% identical to that of B18-123 (Fig. 1d). However, we did not find any information indicating that these strains were associated with severe CDI episodes. The amino acid sequence of B18-123 TcdC showed 95.83% identity with strain R20291 but showed very low identity with strain M120 because of the 39-bp deletion in *tcdC*. Collectively, our results suggest that TcdC carried by B18-123 causes different regulation of TcdA and TcdB production compared to previously identified hypervirulent lineages such as in the NAP1/RT027/ST1, RT019/ST67 (LIBA5757), and RT078/ST11 stains. These findings also indicate that it may be difficult to directly determine the TcdC characteristics of B18-123 by nucleic acid amplification tests available in clinical settings [37].

**Fig. 2:**
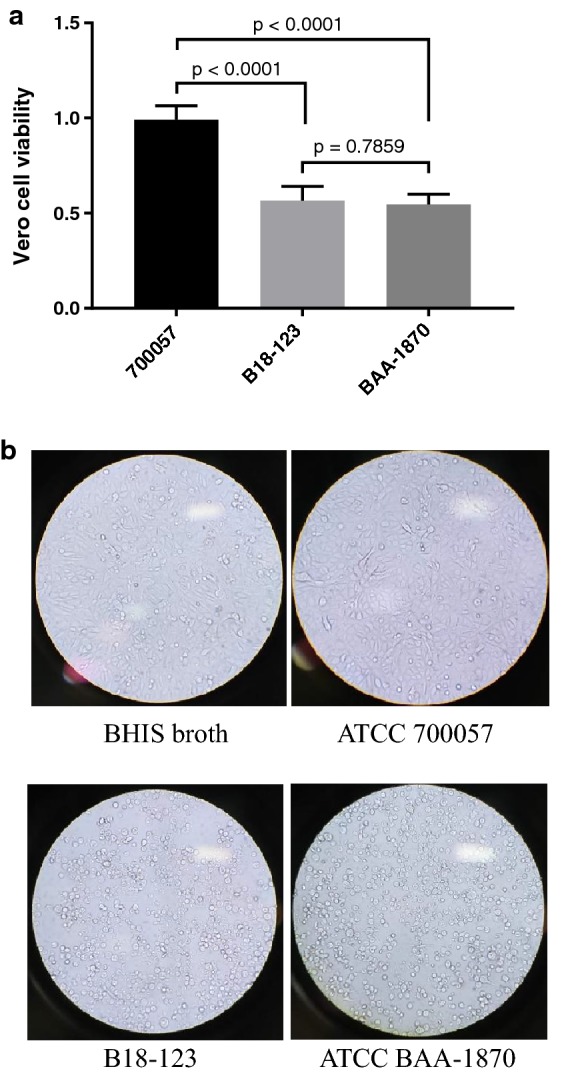
Cytotoxicity of *Clostridioides difficile* strain B18-123 on Vero cells. (**a**) Vero cell viability in B18-123 was evaluated together with positive control ATCC BAA-1870 (NAP1/RT027/ST1; TcdA^+^, TcdB^+^, Cdt^+^) and negative control ATCC 700057 (RT038/ST48; TcdA^−^, TcdB^−^, Cdt^−^) strains, as described previously [16]. The results are presented as means ± standard deviations of cell viability percentages. (**b**) Cytotoxic effect of *C. difficile* strains studied under a light microscope (original magnification × 200)

Although conflicting findings have been reported regarding the functional role of TcdC [38–40], the regulation of TcdA and TcdB production in B18-123, which possesses an Asp7Glu substitution in TcdC, may differ from that in previously identified hypervirulent clade 2 strains. Therefore, we further investigated the relationship between *tcdC* mutation and toxin activity in a cytotoxicity assay [16]. Vero cell viability in B18-123 was similar to that in strain ATCC BAA-1870 (NAP1/RT027/ST1) with an 18-bp deletion and a single-bp deletion at position 117 in *tcdC* (*P* = 0.7859) (Fig. 2a). This result was related to the morphological changes shown by cell rounding of Vero cells observed under a light microscope (Fig. 2b). These findings suggest that TcdC carried by B18-123 allows the cell to increase toxin production, leading to high virulence. However, we could not directly quantify both TcdA and TcdB or clarify the mechanism of this protein in toxin regulation in B18-123 in this study.

As strain B18-123 also possesses *cdtA* and *cdtB*, CdtLoc (5.2 kb) carried by B18-123 was compared to those of the R20291 and M120 strains. The BLAST results indicated that CdtLoc in B18-123 showed homology with the R20291 (100% query cover and 99.90% identity) and M120 (100% query cover and 97.99% identity) strains. The binary toxin regulator gene *cdtR* (747 bp) in B18-123 was completely conserved with that in strain R20291 (100% query cover and 100% identity) and highly conserved with that in strain M120 (100% query cover and 97.46% identity), indicating that CdtR carried by B18-123 modulates binary toxin production similar to in NAP1/RT027/ST1 strains.

PHASTER identified three complete prophage sequences (scores > 90 and over; Table 1) and showed that strain B18-123 possessed the *C. difficile* bacteriophages phiMMP02, phiCDHM19 (belong to Myoviridae family), and phiCD111 (belong to Siphoviridae family). Although the B18-123 genome also contained one questionable and three incomplete prophage sequences, further studies are needed to understand the role of the phages in the evolution of RT019/ST67 lineages.

**Table 1:** Summary of phage-related regions identified by PHASTER in the B18-123 genome

Region	Length of prophage (kb)	Completeness	PHASTER score	No. of total proteins	Region position	Most common phage	GC content (%)	Location on scaffolds
1	69.2	Intact	150	93	294078–363300	PHAGE_Clostr_phiMMP02_NC_019421 (26)	28.18	NODE001
6	44.8	Intact	140	66	1–44849	PHAGE_Clostr_phiCDHM19_NC_028996 (21)	28.25	NODE021
7	40.4	Intact	125	48	48–40472	PHAGE_Clostr_phiCD111_NC_028908 (46)	30.88	NODE022

All contigs were merged into a single continuous sequence on PHASTER (http://phaster.ca/). B18-123 genome also contained three incomplete and one questionable prophage sequences (scores < 90)

Strain B18-123 was susceptible to metronidazole (MIC = 0.19 mg/L), vancomycin (MIC = 0.75 mg/L), rifampin (MIC ≤ 0.002 mg/L), and moxifloxacin (MIC = 1.0 mg/L). Additionally, B18-123 showed low-level MICs to erythromycin (1.5 mg/L) and clindamycin (0.75 mg/L). We then performed in silico analysis to investigate the presence of antibiotic resistance genes using ResFinder version 3.1 [21], ARG-ANNOT [22], and CARD [23]. We detected only a C656T nucleotide substitution within the 23S rRNA copy on the genome with CARD. However, as a previous study suggested, the substitution has been not associated with conferring resistance to macrolides and lincosamides in *C. difficile* [41]. Our findings obtained from the resistome analysis seem to be sound and coincide with phenotypic results of antibiotic MICs. As evidenced by our MIC results with vancomycin on B18-123, treatment with the same was very effective for treating the CDI in this patient. Additionally, no amino acid substitutions in GyrA or GyrB were identified between B18-123 and fluoroquinolone-susceptible *C. difficile* strain 630 [42]. These results agree with those of a previous study of RT019/ST67 strain [10].

## Conclusion

In conclusion, we reported for the first time that *C. difficile* strain B18-123 in Japan belongs to hypervirulent clade 2, RT019/ST67. Our findings enhance our understanding of the characteristics of hypervirulent clade 2 strains and further emphasize the need for continuous monitoring of *C. difficile* isolates to assess the spread of clade 2 strains in Japan.

## Data Availability

This whole-genome shotgun project has been deposited at DDBJ/EMBL/GenBank under the Bioproject number PRJNA530817.

## Abbreviations

RT: ribotype
MLST: multilocus sequence typing
ST: sequence type
TcdA: toxin A
TcdB: toxin B
Cdt: binary toxin
PaLoc: pathogenicity locus
CdtLoc: Cdt locus
MIC: minimum inhibitory concentration
